# Different Plasma Markers of Inflammation Are Influenced by Immune Recovery and cART Composition or Intensification in Treated HIV Infected Individuals

**DOI:** 10.1371/journal.pone.0114142

**Published:** 2014-12-02

**Authors:** Marta Massanella, Dan Ouchi, Silvia Marfil, Josep M. Llibre, Maria C. Puertas, María J. Buzón, Douglas D. Richman, Elisa Orna, Mario Stevenson, Josep M. Gatell, Pere Domingo, Eugènia Negredo, Javier Martinez-Picado, Bonaventura Clotet, Julià Blanco

**Affiliations:** 1 AIDS Research Institute IrsiCaixa-HIVACAT, Institut d’Investigació en Ciències de la Salut Germans Trias i Pujol, UAB, Badalona, Spain; 2 VA San Diego Healthcare System and Departments of Pathology and Medicine, University of California San Diego (UCSD), San Diego, United States of America; 3 “Lluita contra la SIDA” Foundation, University Hospital Germans Trias i Pujol, Institut d’Investigació en Ciències de la Salut Germans Trias i Pujol, Badalona, Spain; 4 Hematology Department, University Hospital Germans Trias i Pujol, Badalona, Spain; 5 University of Miami, Miami, United States of America; 6 Hospital Clínic-IDIBAPS, Barcelona, Spain; 7 Hospital de Sant Pau, Barcelona, Spain; 8 Universitat de Vic-UCC, Vic, Spain; 9 Institució Catalana de Recerca i Estudis Avançats (ICREA), Barcelona, Spain; University of Nebraska Medical Center, United States of America

## Abstract

**Background:**

HIV-1 infection increases plasma levels of inflammatory markers. Combination antiretroviral therapy (cART) does not restore inflammatory markers to normal levels. Since intensification of cART with raltegravir reduced CD8 T-cell activation in the Discor-Ral and IntegRal studies, we have evaluated the effect of raltegravir intensification on several soluble inflammation markers in these studies.

**Methods:**

Longitudinal plasma samples (0–48 weeks) from the IntegRal (n = 67, 22 control and 45 intensified individuals) and the Discor-Ral studies (44 individuals with CD4 T-cell counts<350 cells/µl, 14 control and 30 intensified) were assayed for 25 markers. Mann-Whitney, Wilcoxon, Spearman test and linear mixed models were used for analysis.

**Results:**

At baseline, different inflammatory markers were strongly associated with HCV co-infection, lower CD4 counts and with cART regimens (being higher in PI-treated individuals), but poorly correlated with detection of markers of residual viral replication. Although raltegravir intensification reduced inflammation in individuals with lower CD4 T-cell counts, no effect of intensification was observed on plasma markers of inflammation in a global analysis. An association was found, however, between reductions in immune activation and plasma levels of the coagulation marker D-dimer, which exclusively decreased in intensified patients on protease inhibitor (PI)-based cART regimens (*P* = 0.040).

**Conclusions:**

The inflammatory profile in treated HIV-infected individuals showed a complex association with HCV co-infection, the levels of CD4 T cells and the cART regimen. Raltegravir intensification specifically reduced D-dimer levels in PI-treated patients, highlighting the link between cART composition and residual viral replication; however, raltegravir had little effect on other inflammatory markers.

## Introduction

Human immunodeficiency virus type 1 (HIV-1) infection leads to immune activation that has been related to massive CD4 T-cell depletion occurring mainly in the gut associated lymphoid tissue (GALT), which in turn favors the translocation of bacterial products from the intestinal lumen to the circulation and tissues [1]. In addition, a chronic Type 1 interferon (IFN) response driven by HIV-1 replication, co-infecting pathogens and the homeostatic proliferation, attempting to counterbalance CD4 T-cell depletion have been also reported as potential sources of increased immune activation [2]–[5]. Cellular markers of immune activation, such as the turnover or the expression of activation and maturation markers in CD4 or CD8 T cells have been commonly used to determine the level of immune damage in HIV-1 infection [6]–[8]. Soluble markers of inflammation can be also used as biomarkers for immune activation or antiviral responses, and have been widely employed to fully delineate the aberrant immune activation observed in HIV infected individuals [9]–[11].

Combination antiretroviral therapy (cART) effectively suppresses viral replication and in most cases increases CD4 T-cell counts, but only partly reduces T-cell activation, cell death and soluble inflammatory markers [7], [12]–[15]. This observation is clinically relevant since many authors have associated increased levels of inflammatory, coagulation or endothelial function markers in cART treated individuals to several clinical outcomes, including death [16], subclinical cardiovascular risk [17], [18], AIDS defining events or non-AIDS defining events [19],[20]. The reasons for this incomplete effect of cART in immune restoration are still elusive. Candidate factors could include an incomplete CD4 T-cell repopulation [12], [21] and HIV-1 persistence, defined by the presence of HIV-1 antigens coming from long-lived reservoirs or from low level HIV-1 replication in sanctuary sites [22], [23].

Intensification of cART regimens has been used as a strategy to contain residual HIV-1 replication in virologically suppressed individuals [24]–[28]. Although the reported virological and immunological results are controversial [24], [25], [27], [28], a relevant proportion of intensified individuals showed evidence of an effective blockade of new infection events as measured by the presence of episomal viral DNA (2-long terminal repeat [2-LTR] circles) that was accompanied by a decrease in some markers of immune activation or inflammation [24], [25].

The IntegRal [24], [26], [29] and Discor-Ral [30], [31] studies explored the effect of intensification of cART with the integrase inhibitor raltegravir in virologically suppressed individuals. While the IntegRal study focused on patients with proper immune recovery, the Discor-Ral study investigated patients with unsatisfactory immune recovery (defined by a CD4 T-cell count below 350 cells/µL). Both studies offered an opportunity to address the contribution of incomplete immune recovery and low level HIV-1 replication under cART to the inflammatory status of virologically suppressed HIV-infected individuals.

We analyzed inflammation in baseline samples from these studies to evaluate the association of inflammation and incomplete immune recovery. In addition longitudinal analysis was used to test the effect of the blockade of low level HIV-1 replication under cART in the inflammatory status of virologically suppressed HIV-infected individuals. In order to cover a large number of potential markers, we evaluated a collection of 25 cytokines in baseline and longitudinal samples from both studies. We included pro-inflammatory mediators of innate immunity (i.e. TNF-α, IL-1b, IL-6, IL-10, IL-12, IL-15, type I interferon, such as IFN-α, α- and β-chemokines, such as IL-8, MCP-1 and MIP-1β) or adaptive immunity (i.e. IL-2, IL-4, IL-5, IL-13, IL-17 and IFN-γ), pro-apoptotic inductors (TRAIL), stimulators of hematopoiesis (i.e. GM-CSF and G-CSF), monocyte activation (sCD14), endothelium inflammation (sICAM and sVCAM) and hypercoagulation markers (D-dimer, a fibrin degradation product that can be increased with inflammation). Our data suggest a link between systemic inflammatory markers and CD4 T-cell recovery and also with the composition of cART regimens. Indeed, a specific beneficial effect of raltegravir intensification could be identified in individuals receiving protease inhibitor (PI)-based regimens.

## Materials and Methods

### Ethics statement

The Discor-Ral study is a prospective, open-label, randomized study (Clinical-Trials.gov number NCT00773708) to assess the effect of raltegravir addition to standard antiretroviral therapy for 48 weeks in immunodiscordant individuals (Discor-Ral Study) carried out in the Hospital Universitari Germans Trias i Pujol (Badalona, Spain) [30], [31]. The IntegRal study is a prospective, controlled, open-label study that includes HIV-1-infected subjects on suppressive HAART for at least 1 year (ClinicalTrials.gov number NCT00554398) carried out in three university hospitals in Barcelona [24], [26], [29]. The Ethics Committee of the Hospital Germans Trias i Pujol approved The Discor-Ral Study, while the IntegRal study was approved by the Ethics Committee of the Hospital Germans Trias I Pujol (Badalona, Spain), the Ethics Committee of the Hospital Clinic (Barcelona, Spain) and the Ethics Committee of the Hospital de Sant Pau (Barcelona, Spain). All participants provided written informed consent to participate in these studies.

### Samples

**Figure 1:**
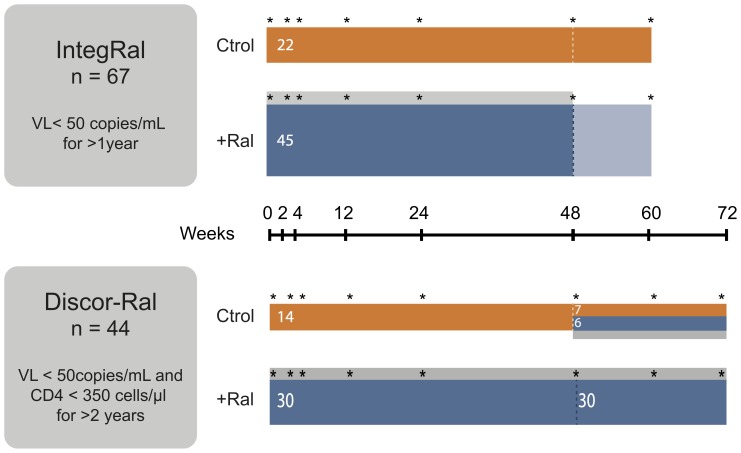
Schematic representation of IntegRal and Discor-Ral studies. In both studies, enrolled individuals were randomized 1∶2 to continue with previous cART (Ctrol, control groups in orange) or intensify their therapy with raltegravir (+Ral, intensified groups in blue). The extent of raltegravir intensification was 48 weeks for the IntegRal study and 72 weeks for the Discor-Ral study (grey areas). The follow up of the IntegRal study was 60 weeks, allowing for the analysis of the effect of raltegravir discontinuation. In the Discor-Ral study six patients from the control arm started raltegravir for 24 weeks (blue area). In all groups, asterisks denote sampling times.

Plasma samples were obtained from EDTA-treated blood, aliquoted and stored at –80°C at specified time points during the IntegRal and the Discor-Ral studies (Figure 1). Samples from the IntegRal study cover the full intensification period of 48 weeks [26] and an additional 12-week period after raltegravir discontinuation, to assess the stability of the effect of the drug [29]. Samples from the Discor-Ral study included longer intensification periods (up to 60 weeks), but lack additional samples after raltegravir discontinuation [31]. Inclusion criteria were similar for both studies, except that the IntegRal study had no enrollment restriction in terms of CD4 T-cell counts [26], while the Discor-Ral study was restricted to patients with CD4 T-cell counts below 350 cells/µl during the previous two years [30], [31]. In both studies, HIV-1-infected subjects were eligible if they were 18 years of age or older, received a cART regimen composed of two nucleos(t)ide reverse transcriptase inhibitors (NRTIs), and a protease inhibitor (PI) or a non-NRTI (NNRTI), and were naïve to integrase inhibitors. Their plasma HIV-1 RNA levels had to be below 50 copies/mL at baseline and during at least the previous year for the IntegRal or two years in the Discor-Ral study with more than 4 determinations. In both studies, subjects were randomly assigned 2∶1 to add raltegravir (400 mg twice daily) or continue their cART regimen.

### Quantification of soluble markers

Frozen plasma samples were thawed and assayed for the levels of different cytokines and inflammation mediators using Luminex technology. Plasma samples were analyzed (dilution ¼) using a Bio-Plex Pro Human Cytokine panel (BioRad, Hercules, CA) that included the determination of the following human cytokines: MIP-1β, IP-10, TRAIL, TNF-α, IFN-γ, IFN-α, G-CSF, GM-CSF, MCP-1, IL-1b, IL-2, IL-4, IL-5, IL-6, IL-7, IL-8 IL-10, IL-12, IL-13, IL-15 and IL-17. For the determination of soluble VCAM-1 (sVCAM-1) and soluble ICAM-1 (sICAM-1) plasma samples were diluted 1/200 and tested using a BioPlex pro human cytokine 2-plex also from BioRad. Plasma levels of D-dimer were analyzed using the HemosIL D-Dimer HS 500 quantitative kit in an ACL TOP equipment (both from Instrumentation Laboratory, Bedford, MA). Plasma levels of soluble CD14 (sCD14) were analyzed as previously described [26], [30] using a commercial ELISA kit (Diaclone, Besançon, France).

### Statistical analysis

Correlation analysis of soluble plasma markers with immune activation, CD4 T-cell counts and other variables at baseline was performed; however, the primary endpoint was the assessment of changes of soluble plasma biomarkers at week 48 in raltegravir intensified patients. Secondary endpoints were the analysis of changes at intermediate time points using linear mixed models, and the analysis of changes 12 weeks after raltegravir discontinuation (IntegRal study). We performed separate analyses for IntegRal and Discor-Ral studies and a combined analysis aimed to define common effects of intensification in both studies; corrections for CD4 T-cell counts, HCV status or treatment regimen were performed.

We used the Mann-Whitney U test to compare medians between different arms or different studies and the signed-rank test (paired test) to compare longitudinal differences within groups (baseline-week 48, and week 48-week 60). The relationship between soluble plasma markers and different co-variables was examined using the nonparametric Spearman test. Regression coefficients and *p*-values for each cytokine are reported from a multivariate linear regression model: *cytokine = intercept+HCV status +2LTR detection+age+PI/NNRTI based cART+nadir+CD4 counts+gender*. Specifically, the final multivariate model was derived using a bootstrapped AIC based stepwise selection method. Moreover, the study of longitudinal changes within groups and the mean change comparison between arms (slope coefficient), from baseline to week 48, were evaluated using linear mixed models: cytokine = intercept+group+week+group*week. A statistically significant interaction term (group*week) identified significantly different slopes among treatment groups. All analyses were done using the R package (v 3.0.2) [32], [33].

## Results

### Baseline characteristics of patients enrolled in the IntegRal and Discor-Ral studies

The individuals enrolled in the IntegRal and Discor-Ral studies are described in Table 1. No major differences were observed at baseline among control and intensified groups in each study, except for the previously reported higher prevalence of hepatitis C virus (HCV) infection in the control group of the Discor-Ral study [30], [31]. As expected, participants in the Discor-Ral study had lower nadir and baseline CD4 T-cell counts than participants in the IntegRal study, but also were significantly older, were infected longer and had a higher percentage of PI-based regimens (Table 1).

**Table 1:** Baseline characteristics of the control and raltegravir-intensified individuals.

		IntegRal			Discor-Ral		*P-value*
	Control	Intensified	*P-value*	Control	Intensified	*P-value*	*Between*
	n = 22	n = 45	*Between*	n = 14	n = 30	*Between*	*Studies ^b^*
			*Groups ^a^*			*Groups ^a^*	
**Age**, years, Median [IQR]	45 [37–50]	44 [41–49]	*0.569*	48 [42–51]	45 [44–53]	*0.221*	***0.007***
**Females** n (%)	6 (27.3)	6 (9.0)	*0.187*	3 (13.3)	4 (14.3)	*0.932*	*1*
**Hepatitis C co-infection**, n (%)	5 (22.7)	13 (28.1)	*0.810*	7 (50.0)	6 (20.0)	***0.005***	*0.830*
**PI-based ART at Baseline**, n (%)	8 (36.4)	14 (31.1)	*0.878*	8 (57.1)	18 (60.0)	*0.878*	***0.010***
**Time from HIV-1 diagnosis**,years, Median [IQR]	10 [5]–[15]	11 [8]–[17]	*0.795*	15 [5]–[20]	14 [7]–[21]	*0.678*	***0.020***
**Time with suppressive (VL<50)** **ART**, years, Median [IQR]	5 [3]–[8]	7 [3]–[7]	*0.311*	6 [4]–[10]	6 [4]–[9]	*0.868*	*0.070*
**CD4 T cell at baseline,**(cell/µl), Median [IQR]	503[371–600]	530[434–678]	*0.333*	242[188–292]	253[208–301]	*0.668*	***<0.0001***
**Nadir CD4 T-cell count,**(cell/µl), Median [IQR]	84[41–254]	251[85–405]	*0.060*	67[14–108]	90[24–117]	*0.435*	***<0.0001***

PI, protease inhibitors; ART, antiretroviral treatment; VL, plasma viral load (HIV-1 RNA copies/ml);

a p-value between groups: Mann-Whitney U test,

b p-value of the comparison of patients enrolled in the IntegRal (n = 67) and DIscor-Ral (n = 44) studies: Mann-Whitney U test.

**Figure 2:**
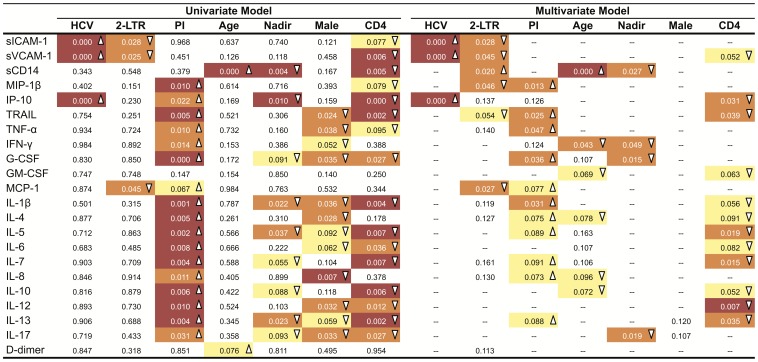
Analysis of baseline levels of soluble plasma markers. Heat map of the association of baseline levels of the different soluble plasma markers analyzed with the following parameters: HCV co-infection (HCV column), presence of 2-LTR during the study period (2-LTR column), the presence of PIs on the cART regimen (PI column, PI = 1, NNRTI = 0), age, Nadir CD4 T-cell counts (nadir column), gender (male column, male = 1, female = 0) and CD4 T-cell counts at study baseline (CD4 columns). Left part shows results from a univariate analysis (spearman test), while right side data correspond to a multivariate analysis carried out using the AIC-based stepwise procedure. In all cases, color codes are as follow: no color means *p*-value>0.1, yellow 0.1>*p*-value>0.05; orange 0.05>*p*-value>0.01 and red *p*-value<0.01. Upward arrowheads (Δ) indicate positive correlations while downward arrowheads indicate negative correlations.

All plasma markers examined were detectable in most samples, except for IL-2, IL-15 and IFN-α, which were undetectable in>80% of plasmas tested, hampering a proper analysis of these cytokines. A comparison of detectable soluble plasma markers among control and intensified groups in each study at baseline showed no major differences (data not shown). However, when the IntegRal and Discor-Ral studies were compared at baseline, significantly higher levels of most markers were noticed in the latter study (Figure S1). Since several factors could account for these differences (including lower CD4 T-cell counts and cART composition), we analyzed the association of the levels of soluble plasma markers with demographic, immunological and virological factors (Figure 2). Univariate analysis showed some relationship of soluble markers with age, the detection of 2-LTR circles during intensification and with HCV co-infection, while inflammatory status appeared to be more related to sex, cART composition (only when PI-based *vs* NNRTI-based regimens were compared), nadir and current CD4 T-cells values. A multivariate analysis maintained strong associations between HCV infection and levels of IP-10, sICAM-1 and sVCAM-1, between age and sCD14 and an inverse association between CD4 T-cell counts and IL-12 (Figure 2, red boxes). Moreover, the presence of 2-LTR circles, the administration of PI-containing regimens, CD4 T-cell counts, the CD4 nadir, and age were also independently associated with several inflammatory markers (Figure 2, orange boxes). However, while markers of inflammation showed positive and negative associations with the detection of 2-LTR circles, a more consistent scenario was observed for other parameters: higher plasma levels were identified in patients on a PI-based cART (positive correlations) and in individuals with low CD4 T-cell counts or with low nadir CD4 T cell values (negative correlations). Interestingly, sCD14 was the marker with the highest number of significant associations: 2LTR detection, age and nadir CD4 T-cell counts.

### Effect of raltegravir intensification

**Figure 3:**
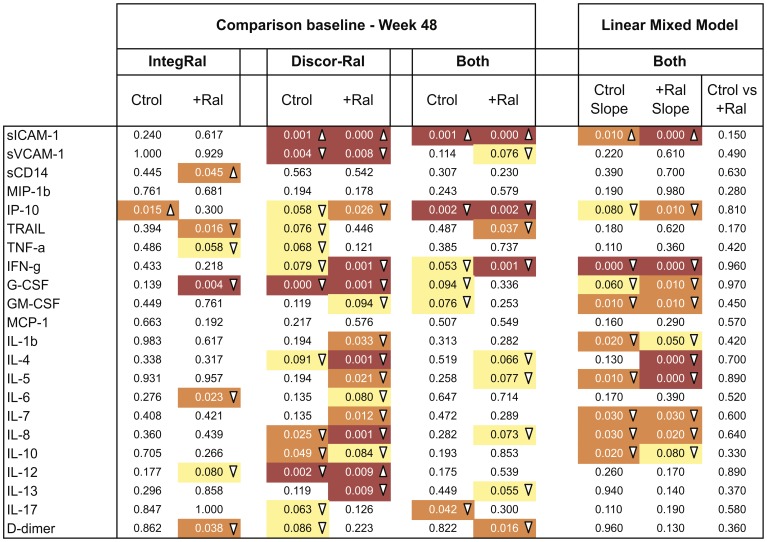
Longitudinal analysis of soluble plasma markers. Left part shows the heat map of the *p*-values obtained by comparing the levels of plasma soluble markers at baseline and week 48 in the IntegRal and Discor-Ral studies or in a global analysis of both studies. *P*-values are given for the control (Ctrol) and intensified (+ Ral) groups as indicated. Right part shows the results from a linear mixed model analysis showing *p*-values for slope (change of every marker per week) for control and intensified groups and the interaction slope/group that indicates the existence of significant difference among groups. As in Figure 2, color codes are as follow: no color means *p*-value>0.1, yellow 0.1>*p-*value>0.05; orange 0.05>*p*-value>0.01 and red *p*-value<0.01. Upward arrowheads (Δ) indicate positive correlations while downward arrowheads indicate negative correlations.

Longitudinal analyses of soluble plasma markers were performed by comparing baseline and week 48 data and by fitting linear mixed models that included intermediate time points (Figure 1), as reported for the evolution of CD4 T cells or immune activation markers [26]. The direct comparison of baseline and week 48 levels of plasma soluble markers showed minimal changes in the IntegRal study. Only D-dimer, G-CSF, IL-6 and TRAIL showed significant decreases in the intensified group that were not observed in control individuals (Figure 3). In contrast, the Discor-Ral study showed a more pronounced effect of raltegravir intensification upon cytokines including IFN-γ, IL-13, IL-1b, IL-4, IL-5, IL-7 and IP-10, probably due to higher baseline values. Since changes were not fully consistent in both studies, we explored potential common effects of raltegravir by performing a combined analysis of both studies. This combined analysis showed specific changes induced by raltegravir in D-dimer, IFN-γ and TRAIL in intensified, but not in control individuals, with trends for IL-4, IL-5, IL-8 and IL-13. A more powerful analysis using linear mixed models that included all longitudinal data was also applied (Figure S2). This approach detected significant decreases of IP-10, G-CSF, GM-CSF, IFN-γ, IL-1b, IL-4, IL-5, IL-7 and IL-8 but failed to show significant differences among slopes calculated in control and intensified groups for these cytokines (Figure 3, right columns). A significant increase of sICAM-1, was seen in both the raltegravir and control groups (Figure 3). Similar results were observed when analyses were performed including corrections for CD4 T-cell counts, HCV status or treatment regimen (data not shown). Analysis of plasma markers 12 weeks after raltegravir discontinuation in the IntegRal study showed no statistically significant changes compared to week 48 values in any marker.

### Association of changes in immune activation and soluble markers

We previously reported that raltegravir intensification of treated subjects with suppressed viremia induced a specific decrease in markers of immunological activation in CD8 T cells, particularly for CD38 [26], [30]. Thus, we explored in the current study whether these changes were associated with soluble markers of inflammation. In this analysis, no significant association between CD8 T-cell activation, as measured by CD38 expression, and inflammatory markers were observed. However, a trend for association with the levels of D-dimer (*p*-value = 0.09) was seen, which was stronger in the IntegRal study (*p*-value = 0.07, data not shown).

**Figure 4:**
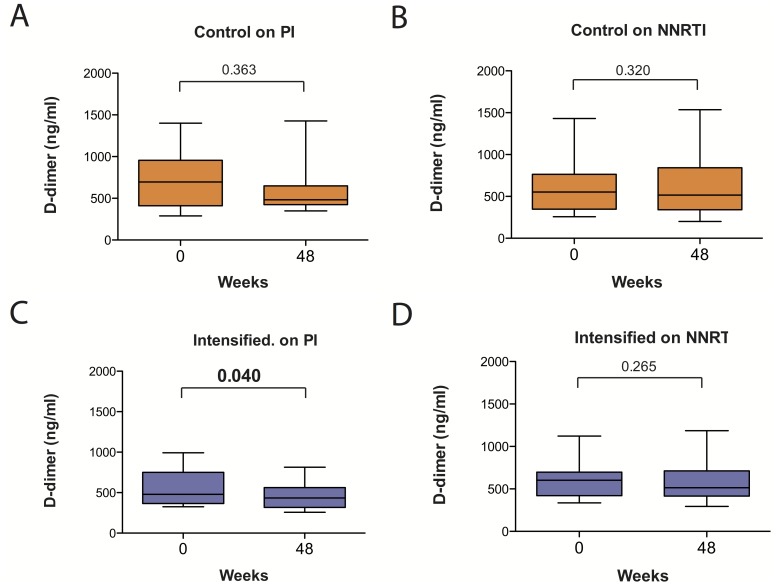
Longitudinal changes in plasma level of D-dimer according to cART regimen. Individuals from both the IntegRal and Discor-Ral studies were classified according to their cART regimen, PI- or NNRTI-based. The level of D-dimer in plasma at baseline and after 48 weeks of study was compared. The figure shows changes for Control individuals on PI-based regimens (A, n = 15), control individuals on NNRTI-based regimens (B, n = 17), raltegravir-intensified individuals on PI-based regimens (C, n = 27) and raltegravir-intensified individuals on NNRTI based regimens (D, n = 33). Comparisons were performed using the signed-rank test (paired test) and p-values are shown for each group.

To better understand this partial association, we have classified individuals undergoing treatment intensification according to the detection of 2-LTR circles with raltegravir intensification or to the presence of a PI in their cART regimen, which has been also related to the detection of 2-LTR circles [24], [25]. No significant changes in plasma levels of D-dimer were observed when these individuals were grouped according to the detection of 2-LTR circles (data not shown). However, a significant decrease was observed in patients treated with a PI-based regimen in the IntegRal study (*p-*value = 0.045). Although a similar comparison in the Discor-Ral study did not achieve statistical significance, the combined analysis of both studies confirmed a significant effect of raltegravir on the levels of D-dimer in PI-treated patients (*p-*value = 0.040), that was not observed in raltegravir intensified individuals on NNRTI therapy or in control individuals irrespective of their cART regimen (Figure 4). Consistent with the small changes in inflammatory markers induced by raltegravir intensification, no major increase in inflammation was noticed after raltegravir discontinuation (data not shown), as reported for immune activation in the IntegRal study [29].

## Discussion

The impact of raltegravir intensification of suppressive cART regimens on soluble plasma markers might provide new clues regarding the contribution of residual viral replication to the subclinical inflammatory status of treated HIV-1 infected individuals. Our analysis of two separate studies (IntegRal and Discor-Ral) points to a poor association between plasma inflammatory markers and the detection of residual viral replication (assessed by 2-LTR circles) in treated individuals with chronic HIV infection. Instead, we have identified a complex but strong relationship between these markers and other immunological and virological parameters, including CD4 T-cell restoration.

Although we observed a significant association between several cytokines and the number of circulating CD4 T cells, this observation could be influenced by the higher prevalence of PI-based regimens in individuals with low CD4 T-cell counts, a phenomenon previously observed in different cohorts [14]. A multivariate approach performed to identify independent associations confirmed that some plasma markers (including IL-5, IL-7, IL-12, IL-13, TRAIL or IP-10) were independently associated with the number of CD4 T cells. In addition, this analysis also revealed other factors influencing inflammation. Among them, Nadir CD4 T-cell values that showed independent association with sCD14 and IL-17 levels. And the detection of 2-LTR circles and baseline that showed a more complex association with the levels of inflammation. The interpretation of these data is probably limited by the biased sample selection; namely, a high representation of individuals with low Nadir CD4 T-cell counts (in the Discor-Ral but also in the IntegRal study) and a low number of patients whose mononuclear cells had 2-LTR circles, especially in the Discor-Ral study. New technologies such as droplet digital PCR [34], may help to overcome technical limitations to detect 2-LTR circles and to better identify individuals with residual viral replication [25]. However, the complex relationship of inflammatory markers with several immunological and virological variables may complicate their use as predictors for residual HIV replication under cART.

Indeed, our baseline analysis confirmed the strong association of sCD14 with age [35], the link of HCV co-infection with three soluble markers (IP-10, sICAM-1 and sVCAM-1) [36], and identified a striking association of several cytokines, TRAIL and TNF-α among other, with the cART regimen (PI- or NNRTI-based). In all cases, this latter association revealed higher levels of inflammation in individuals treated with PI-based regimens, an observation that is consistent with previously reported data [11], [18]. However, the mechanisms leading to this increased inflammation are unclear. PI-treated individuals are known to display altered lipid metabolism, resulting in higher levels of plasma lipids and higher cholesterol accumulation in macrophages that may result in higher levels of inflammation and endothelial dysfunction markers in plasma [37]–[39]. In fact, switching PI-based to raltegravir-based cART results in specific reductions of some of these markers [37]. In addition to metabolic interference of PIs, an alternate explanation for the increased inflammation in these patients can be related to the poor tissue penetration of these compounds that may favor low level viral replication [40], [41]. This hypothesis is supported by the higher prevalence of the detection of 2-LTR circles in PI-treated individuals [24], [25], and may also explain the reduction in inflammation induced by switching PI to raltegravir treatment [37]. However, this could suffer a clinical bias, as treating physicians often include PIs in more advanced or immuno-discordant patients.

In our longitudinal analysis raltegravir was added to PI- or NNRTI-based cART and therefore, no metabolic benefits are expected. Despite this, some significant changes were observed in intensified individuals in the Discor-Ral study, which showed higher baseline levels of inflammatory markers. However, a combined analysis of the Discor-Ral and IntegRal studies failed to identify a general decrease in inflammatory markers associated with raltegravir intensification. In fact, several cytokines showed decreasing trends over time in both control and intensified groups. The observation that most of decreasing cytokines (namely IP-10, TRAIL or IL-7) are also associated with lower CD4 T-cell counts suggests that the observed decrease could be the consequence of slow but significant increases in CD4 T-cell counts reported in both the IntegRal and Discor-Ral studies [26], [31]. However, raltegravir induced a significant reduction of the coagulation marker D-dimer that was exclusively observed in PI-treated individuals. D-dimer levels reflect HIV-induced perturbations of the coagulation cascade, contributing to thrombus formation and cardiovascular morbidity [42]. This observation is consistent with data reported by Hatano *et al.* [25] and suggests a specific beneficial effect of raltegravir on PI-treated individuals regarding cardiovascular morbidities. Since no drug switch exists in our study, and the effect is not general to all intensified individuals, it seems to be the consequence of a better *in vivo* antiviral profile of raltegravir rather than a non-specific anti-inflammatory activity of this drug. Consistent with this specific effect, no changes in raltegravir-intensified patients were observed for other inflammatory markers such as IL-6, whose levels have been recently shown to predict non-AIDS-defining morbid events during suppressive HAART [43]. Finally, the reduction in D-dimer levels induced by raltegravir was more evident in individuals with high CD4 T-cell counts (IntegRal study) than in patients with CD4 T-cell counts below 350 (Discor-Ral study), although those were more likely on PI-based regimens. This apparent paradox could be related to the low level of 2-LTR circle detection [30]. Alternatively, the profound immunological abnormalities found in patients with low CD4 T-cell counts that are not reversed by raltegravir [12] [31] may sustain D-dimer levels in these patients.

In summary, our data suggest that soluble inflammatory markers show a complex relationship with several factors (age, co-infections, antiretroviral regimen and immune recovery) that may render the use of these markers to identify individuals with residual viral replication under cART poorly reliable. In fact, the detection of 2-LTR circles is poorly related to inflammation, and raltegravir intensification has only minor effects on inflammatory markers. These observations confirm the minimal clinical effect of intensification in terms of standard follow up (CD4 T-cell counts), but highlight the substantial role of cART composition in inflammation and the detection of 2-LTR circles, emphasizing the need for cART regimens with optimal effects on inflammatory markers that may reduce the risk of clinical events in HIV-1 infected individuals.

## Supporting Information

Figure S1 **Baseline plasma levels of soluble inflammatory markers.** The levels of the indicated soluble markers were assessed prior to raltegravir intensification in patients recruited in the IntegRal (n = 67, blue symbols) or the Discor-Ral (n = 44, red symbols) studies. Control and intensified individuals were grouped for this baseline analysis.
(TIFF)

Figure S2 **Linear models for changes in plasma markers.** Data from the Discor-Ral and IntegRal studies were pooled and analyzed as described in methods (linear mixed models). Longitudinal evolution of CD4 T-cell counts and immune activation (CD38^+^HLA-DR^+^ CD8 T cells) were included for reference (bottom right graphs).
(TIFF)

## References

[1] Brenchley JM, Price DA, Schacker TW, Asher TE, Silvestri G, et al (2006) Microbial translocation is a cause of systemic immune activation in chronic HIV infection. Nat Med 12:1365–1371. 17115046 10.1038/nm1511

[2] Harris LD, Tabb B, Sodora DL, Paiardini M, Klatt NR, et al (2010) Downregulation of robust acute type I interferon responses distinguishes nonpathogenic simian immunodeficiency virus (SIV) infection of natural hosts from pathogenic SIV infection of rhesus macaques. J Virol 84:7886–7891. 20484518 10.1128/JVI.02612-09 PMC2897601

[3] Rotger M, Dalmau J, Rauch A, McLaren P, Bosinger S, et al (2011) Comparative transcriptomics of extreme phenotypes of human HIV-1 infection and SIV infection in sooty mangabey and rhesus macaque. J Clin Invest 121:2391–2400. 21555857 10.1172/JCI45235 PMC3104754

[4] Catalfamo M, Di Mascio M, Hu Z, Srinivasula S, Thaker V, et al (2008) HIV infection-associated immune activation occurs by two distinct pathways that differentially affect CD4 and CD8 T cells. Proc Natl Acad Sci U S A 105:19851–19856. 19060209 10.1073/pnas.0810032105 PMC2596741

[5] Hatano H, Hayes TL, Dahl V, Sinclair E, Lee T-H, et al (2011) A Randomized, Controlled Trial of Raltegravir Intensification in Antiretroviral-treated, HIV-infected Patients with a Suboptimal CD4+ T Cell Response. J Infect Dis 203:960–968. 21402547 10.1093/infdis/jiq138 PMC3068029

[6] Vujkovic-Cvijin I, Dunham RM, Iwai S, Maher MC, Albright RG, et al (2013) Dysbiosis of the gut microbiota is associated with hiv disease progression and tryptophan catabolism. Sci Transl Med 5:193ra91. 10.1126/scitranslmed.3006438 PMC4094294 23843452

[7] Lederman MM, Calabrese L, Funderburg NT, Clagett B, Medvik K, et al (2011) Immunologic failure despite suppressive antiretroviral therapy is related to activation and turnover of memory CD4 cells. J Infect Dis 204:1217–1226. 21917895 10.1093/infdis/jir507 PMC3218674

[8] Appay V, van Lier RAW, Sallusto F, Roederer M (2008) Phenotype and function of human T lymphocyte subsets: consensus and issues. Cytometry A 73:975–983. 18785267 10.1002/cyto.a.20643

[9] Kuller LH, Tracy R, Belloso W, De Wit S, Drummond F, et al (2008) Inflammatory and coagulation biomarkers and mortality in patients with HIV infection. PLoS Med 5:e203. 18942885 10.1371/journal.pmed.0050203 PMC2570418

[10] Strategies for Management of Antiretroviral Therapy (SMART) Study Group, El-Sadr WM, Lundgren JD, Neaton JD, Gordin F, et al (2006) CD4+ count-guided interruption of antiretroviral treatment. N Engl J Med 355:2283–2296. 17135583 10.1056/NEJMoa062360

[11] Cassol E, Misra V, Holman A, Kamat A, Morgello S, et al (2013) Plasma metabolomics identifies lipid abnormalities linked to markers of inflammation, microbial translocation, and hepatic function in HIV patients receiving protease inhibitors. BMC Infectious Diseases 13:203. 23641933 10.1186/1471-2334-13-203 PMC3655873

[12] Massanella M, Negredo E, Clotet B, Blanco J (2013) Immunodiscordant responses to HAART - mechanisms and consequences. Expert Rev Clin Immunol 9:1135–1149. 24168417 10.1586/1744666X.2013.842897

[13] Massanella M, Negredo E, Pérez-Alvarez N, Puig J, Ruiz-Hernández R, et al (2010) CD4 T-cell hyperactivation and susceptibility to cell death determine poor CD4 T-cell recovery during suppressive HAART. AIDS 24:959–968. 20177358 10.1097/QAD.0b013e328337b957

[14] Negredo E, Massanella M, Puig J, Pérez-Alvarez N, Gallego-Escuredo JM, et al (2010) Nadir CD4 T cell count as predictor and high CD4 T cell intrinsic apoptosis as final mechanism of poor CD4 T cell recovery in virologically suppressed HIV-infected patients: clinical implications. Clin Infect Dis 50:1300–1308. 20367229 10.1086/651689

[15] Massanella M, Curriu M, Carrillo J, Gómez E, Puig J, et al (2013) Assessing main death pathways in T lymphocytes from HIV infected individuals. Cytometry A 83:648–658. 23650261 10.1002/cyto.a.22299

[16] Sandler NG, Wand H, Roque A, Law M, Nason MC, et al (2011) Plasma levels of soluble CD14 independently predict mortality in HIV infection. J Infect Dis 203:780–790. 21252259 10.1093/infdis/jiq118 PMC3071127

[17] Deeks SG (2011) HIV infection, inflammation, immunosenescence, and aging. Annu Rev Med 62:141–155. 21090961 10.1146/annurev-med-042909-093756 PMC3759035

[18] Madden E, Lee G, Kotler DP, Wanke C, Lewis CE, et al (2008) Association of antiretroviral therapy with fibrinogen levels in HIV-infection. AIDS 22:707–715. 18356600 10.1097/QAD.0b013e3282f560d9 PMC3156620

[19] Boulware DR, Hullsiek KH, Puronen CE, Rupert A, Baker JV, et al (2011) Higher levels of CRP, D-dimer, IL-6, and hyaluronic acid before initiation of antiretroviral therapy (ART) are associated with increased risk of AIDS or death. J Infect Dis 203:1637–1646. 21592994 10.1093/infdis/jir134 PMC3096784

[20] Unemori P, Leslie KS, Hunt PW, Sinclair E, Epling L, et al (2013) Immunosenescence is associated with presence of Kaposi’s sarcoma in antiretroviral treated HIV infection. AIDS 27:1735–1742. 23435301 10.1097/QAD.0b013e3283601144 PMC4063793

[21] Gazzola L, Tincati C, Bellistrì GM, Monforte A, Marchetti G (2009) The absence of CD4+ T cell count recovery despite receipt of virologically suppressive highly active antiretroviral therapy: clinical risk, immunological gaps, and therapeutic options. Clin Infect Dis 48:328–337. 19123868 10.1086/595851

[22] Massanella M, Martinez-Picado J, Blanco J (2013) Attacking the HIV Reservoir from the Immune and Viral Perspective. Curr HIV/AIDS Rep 10:33–41. 23242702 10.1007/s11904-012-0150-8

[23] Deeks SG, Autran B, Berkhout B, Benkirane M, Cairns S, et al (2012) Towards an HIV cure: a global scientific strategy. Nat Rev Immunol 12:607–614. 22814509 10.1038/nri3262 PMC3595991

[24] Buzón MJ, Massanella M, Llibre JM, Esteve A, Dahl V, et al (2010) HIV-1 replication and immune dynamics are affected by raltegravir intensification of HAART-suppressed subjects. Nat Med 16:460–465. 20228817 10.1038/nm.2111

[25] Hatano H, Strain MC, Scherzer R, Bacchetti P, Wentworth D, et al (2013) Increase in 2-long terminal repeat circles and decrease in D-dimer after raltegravir intensification in patients with treated HIV infection: a randomized, placebo-controlled trial. J Infect Dis 208:1436–1442. 23975885 10.1093/infdis/jit453 PMC3789577

[26] Llibre JM, Buzón MJ, Massanella M, Esteve A, Dahl V, et al (2012) Treatment intensification with raltegravir in subjects with sustained HIV-1 viraemia suppression: a randomized 48-week study. Antivir Ther 17:355–364. 22290239 10.3851/IMP1917

[27] Gandhi RT, Zheng L, Bosch RJ, Chan ES, Margolis DM, et al (2010) The effect of raltegravir intensification on low-level residual viremia in HIV-infected patients on antiretroviral therapy: a randomized controlled trial. PLoS Med 7:e1000321. 20711481 10.1371/journal.pmed.1000321 PMC2919424

[28] Byakwaga H, Kelly M, Purcell DFJ, French MA, Amin J, et al (2011) Intensification of antiretroviral therapy with raltegravir or addition of hyperimmune bovine colostrum in HIV-infected patients with suboptimal CD4+ T-cell response: a randomized controlled trial. J Infect Dis 204:1532–1540. 21930607 10.1093/infdis/jir559

[29] Massanella M, Esteve A, Buzón MJ, Llibre JM, Puertas MC, et al (2013) Dynamics of CD8 T-Cell Activation after Discontinuation of HIV Treatment Intensification. J Acquir Immune Defic Syndr 63:152–160. 23392458 10.1097/QAI.0b013e318289439a

[30] Massanella M, Negredo E, Puig J, Puertas MC, Buzón MJ, et al (2012) Raltegravir intensification shows differing effects on CD8 and CD4 T cells in HIV infected HAART-supressed individuals with poor CD4 T-cell recovery. AIDS 26:2285–2293. 23018435 10.1097/QAD.0b013e328359f20f

[31] Negredo E, Massanella M, Puertas MC, Buzón MJ, Puig J, et al (2013) Early but limited effects of raltegravir intensification on CD4 T cell reconstitution in HIV-infected patients with an immunodiscordant response to antiretroviral therapy. J Antimicrob Chemother 68:2358–2362. 23677919 10.1093/jac/dkt183 PMC4439517

[32] bates D, Maechler M, Bolker B (2012) lme4: Linear mixed-effects models using S4 classes. R package version 0.999999-0. Available: http://CRAN.R-project.org/package=lme4 and http://CRAN.R-project.org/package=lme4.

[33] R Core Team (2012) R: A language and environment for statistical computing. R Foundation for Statistical Computing, Vienna, Austria. ISBN 3-900051-07-0. Available: http://www.R-project.org/ and http://www.R-project.org/.

[34] Strain MC, Lada SM, Luong T, Rought SE, Gianella S, et al (2013) Highly precise measurement of HIV DNA by droplet digital PCR. PLoS One 8:e55943. 23573183 10.1371/journal.pone.0055943 PMC3616050

[35] Reiner AP, Lange EM, Jenny NS, Chaves PHM, Ellis J, et al (2013) Soluble CD14: genomewide association analysis and relationship to cardiovascular risk and mortality in older adults. Arterioscler Thromb Vasc Biol 33:158–164. 23162014 10.1161/ATVBAHA.112.300421 PMC3826541

[36] Roe B, Coughlan S, Hassan J, Grogan A, Farrell G, et al (2007) Elevated serum levels of interferon- gamma -inducible protein-10 in patients coinfected with hepatitis C virus and HIV. J Infect Dis 196:1053–1057. 17763328 10.1086/520935

[37] Martínez E, D’Albuquerque PM, Llibre JM, Gutierrez F, Podzamczer D, et al (2012) Changes in cardiovascular biomarkers in HIV-infected patients switching from ritonavir-boosted protease inhibitors to raltegravir. AIDS 26:2315–2326. 23018438 10.1097/QAD.0b013e328359f29c

[38] Wu X, Sun L, Zha W, Studer E, Gurley E, et al (2010) HIV protease inhibitors induce endoplasmic reticulum stress and disrupt barrier integrity in intestinal epithelial cells. Gastroenterology 138:197–209. 19732776 10.1053/j.gastro.2009.08.054 PMC4644065

[39] Reingold J, Wanke C, Kotler D, Lewis C, Tracy R, et al (2008) Association of HIV infection and HIV/HCV coinfection with C-reactive protein levels: the fat redistribution and metabolic change in HIV infection (FRAM) study. J Acquir Immune Defic Syndr 48:142–148. 18344877 10.1097/QAI.0b013e3181685727 PMC2561207

[40] Fletcher CV, Staskus K, Wietgrefe SW, Rothenberger M, Reilly C, et al (2014) Persistent HIV-1 replication is associated with lower antiretroviral drug concentrations in lymphatic tissues. Proc Natl Acad Sci U S A 111:2307–2312. 24469825 10.1073/pnas.1318249111 PMC3926074

[41] Letendre SL, Capparelli EV, Ellis RJ, McCutchan JA (2000) Indinavir population pharmacokinetics in plasma and cerebrospinal fluid. The HIV Neurobehavioral Research Center Group. Antimicrob Agents Chemother 44:2173–2175. 10898694 10.1128/aac.44.8.2173-2175.2000 PMC90032

[42] Funderburg NT, Mayne E, Sieg SF, Asaad R, Jiang W, et al (2010) Increased tissue factor expression on circulating monocytes in chronic HIV infection: relationship to in vivo coagulation and immune activation. Blood 115:161–167. 19828697 10.1182/blood-2009-03-210179 PMC2808148

[43] Tenorio AR, Zheng Y, Bosch RJ, Krishnan S, Rodriguez B, et al (2014) Soluble markers of inflammation and coagulation, but not T-cell activation, are predictors of non-AIDS-defining morbid events during suppressive antiretroviral treatment. J Infect Dis 210:1248–1259. 24795473 10.1093/infdis/jiu254 PMC4192039

