# Supplementary figures and images for: Different Plasma Markers of Inflammation Are Influenced by Immune Recovery and cART Composition or Intensification in Treated HIV Infected Individuals
Source: PLoS One. 2014 Dec 2;9(12):e114142. doi: 10.1371/journal.pone.0114142 (PMC4252101; doi:10.1371/journal.pone.0114142)

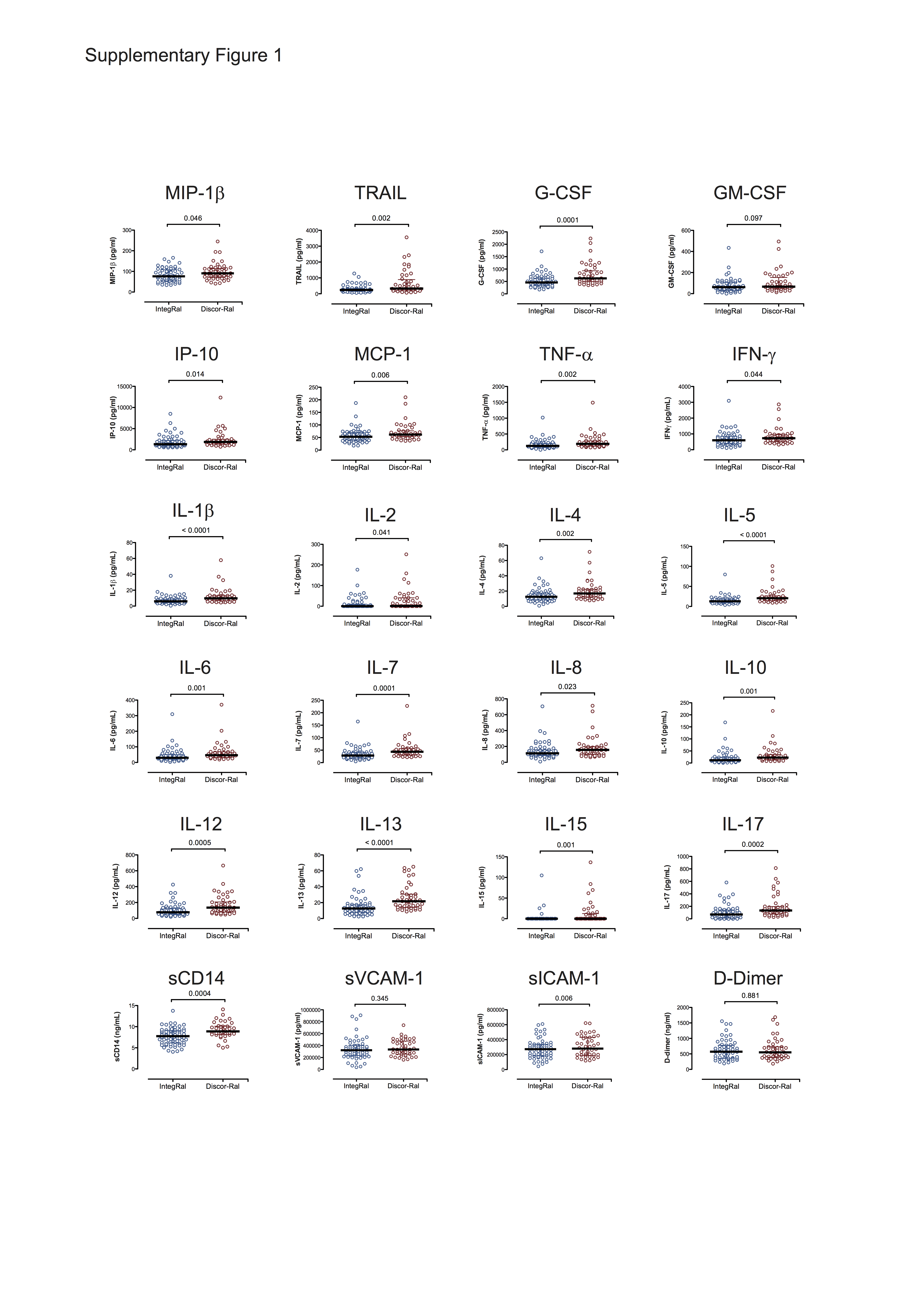

Supplement: Figure S1 — Baseline plasma levels of soluble inflammatory markers. The levels of the indicated soluble markers were assessed prior to raltegravir intensification in patients recruited in the IntegRal (n = 67, blue symbols) or the Discor-Ral (n = 44, red symbols) studies. Control and intensified individuals were grouped for this baseline analysis. (TIFF) [file pone.0114142.s001.tiff]

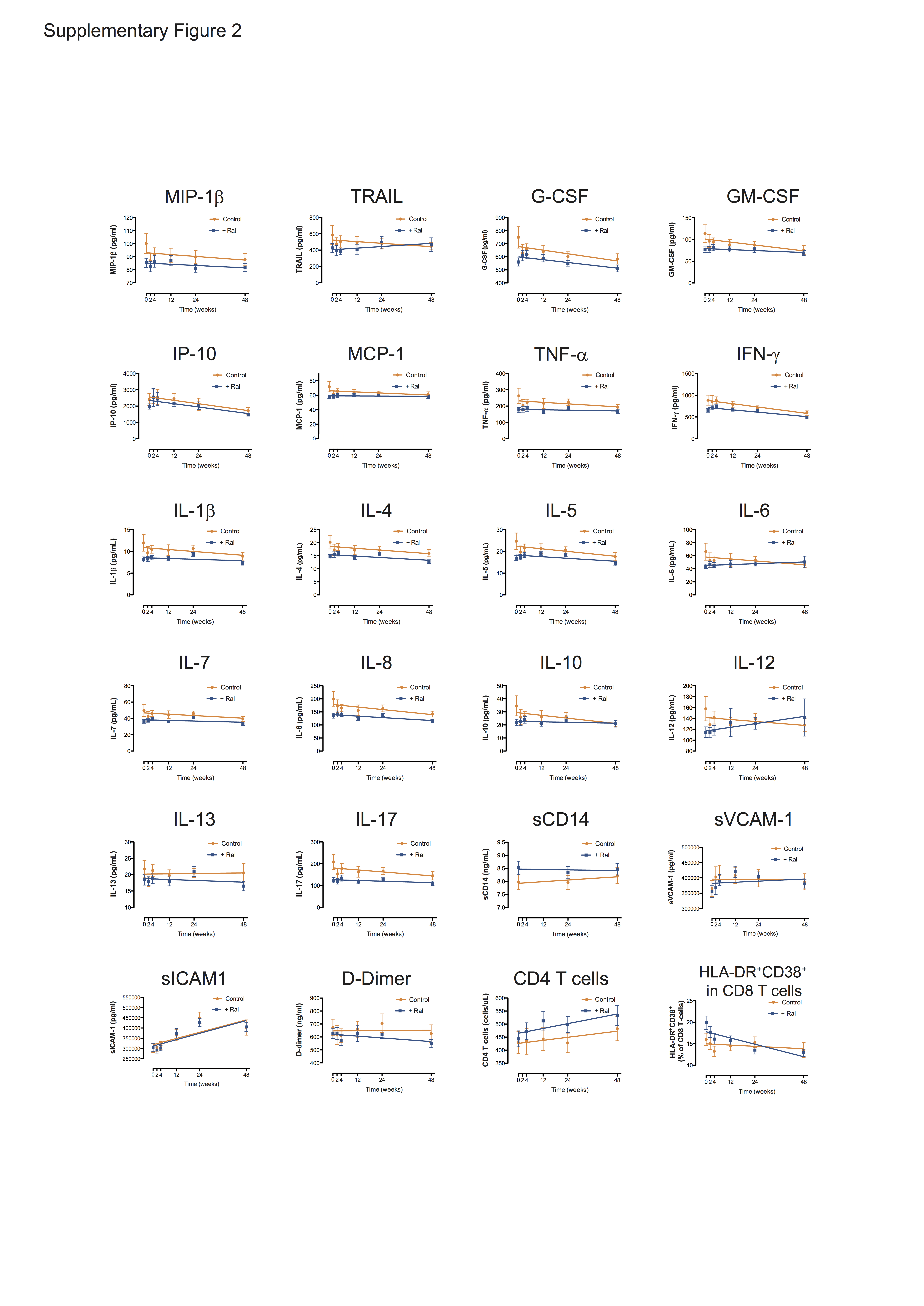

Supplement: Figure S2 — Linear models for changes in plasma markers. Data from the Discor-Ral and IntegRal studies were pooled and analyzed as described in methods (linear mixed models). Longitudinal evolution of CD4 T-cell counts and immune activation (CD38+HLA-DR+ CD8 T cells) were included for reference (bottom right graphs). (TIFF) [file pone.0114142.s002.tiff]
